# Comment on Lundh (2023)

**DOI:** 10.17505/jpor.2024.26273

**Published:** 2024-05-23

**Authors:** S. G. Hofmann, B. Pereira, C. Barbosa, C. E. Lindsay

**Affiliations:** Philipps-University of Marburg, Department of Psychology, Translational Clinical Psychology Group, Marburg, Germany

**Keywords:** psychotherapy, person-level, idiographic design, process-based approach

## Abstract

In this commentary of Lundh’s (2023) article, we point to an individualized process-based approach for the future of psychotherapy. The traditional nomothetic research paradigm is limiting our understanding of processes of change, oversimplifying psychological phenomena, and neglecting individual dynamics. In contrast, a process-based approach calls for ideographic methodologies, departing from the latent-disease paradigm toward process-based interventions. Process-based research promises avenues for enhancing intervention science and a deeper comprehension about psychopathology and therapeutic mechanisms, in a comprehensive, personalized, and holistic manner.

Lundh’s (2023) article entitled, *Person, Population, Mechanism. Three Main Branches of Psychological Science* posits that psychological science has three main branches, corresponding to three different levels of research: *population*-level research, *mechanism*-level research, and *person*-level research. The article makes the convincing case that the failure to differentiate clearly between research questions belonging to these three levels lead to questionable research practices. Therefore, it is of vital importance to differentiate clearly between *change processes at the person-level* and *change processes at the mechanism level* (see for example, Lundh, 2023, p. 85).

In this commentary we elaborate on this issue. Together with Steve C. Hayes, one of us (SGH) developed a new approach toward therapeutic change that builds on processes of change (Hofmann & Hayes, 2019). Hereby, we distinguish the underling therapeutic processes from the therapeutic procedures that are utilized in therapy. Therapeutic procedures include the various strategies or techniques that a therapist uses to achieve the desirable and mutually agreed-upon treatment goals based on measurable outcomes. In contrast, therapeutic processes are the underlying change mechanisms leading to the desirable treatment goals. We (Hofmann & Hayes, 2019) define a therapeutic process

as a set of theory-based, dynamic, progressive, and multilevel changes that occur in predictable empirically established sequences oriented toward the desirable outcomes. These processes are theory–based and associated with falsifiable and testable predictions; they are dynamic, because processes may involve feedback loops and non-linear changes; they are progressive in the long-term in order to be able to reach the treatment goal, they form a multilevel system, because some processes supersede others. Finally, these processes are oriented toward both immediate and long-term goals (p. 38).

For the remainder of this commentary, we will focus on therapeutic processes, rather than the therapeutic procedures and strategies that act on or initiate these processes. A review of some evidence-based procedures can be found elsewhere (Klepac et al., 2012). The term therapeutic process can include the usual meaning as the patient-therapist relationship incorporating common factors (e.g., the therapeutic alliance and other factors of the therapeutic relationship). However, our definition is broader and more tightly connected to empirical evidence based on the psychopathology literature. According to this literature, mechanisms are based on nomothetically derived models of psychology. Although they have advanced treatment efficacy to some extent (Cuijpers et al., 2014; Hofmann et al., 2012; Velthorst et al., 2015), it appears that we might have reached an upper ceiling as shown by stagnating or even decreasing effect sizes over the years.

One of the reasons, we believe, is the over-reliance on a nomothetic approach to mental health and treatment. More specifically, contemporary psychiatric nosology assumes the validity of the latent disease model, postulating that patients’ presented problems are an expression of an underlying, unobserved latent construct, which have resulted in the observed trend of designing specific protocol from syndromes (Hofmann, 2022). The influence of the latent-disease paradigm and its research methodological choices have negatively impacted the field, hindering our knowledge progression, mainly regards processes of change (Hofmann, 2022). On the contrary, endeavors to transition towards transdiagnostic approaches are growing; nevertheless, such efforts remain transitional, as the latent disease entity concept persists (Hofmann, 2022; Hofmann & Hayes, 2019).

The latent disease paradigm assumes that patients’ difficulties and symptoms are independent reflections of unobserved constructs. On the contrary, what is observed is that the patient’s symptomatology forms complex relationships with each other, which can be described as a complex network (Hofmann, 2022).

Likewise, the emergence of an individualized process-based approach as a paradigm shift is central in mental health intervention, allowing for a progressive pathway to a deeper and more comprehensive understanding of mental health difficulties and psychotherapy efficacy (Hofmann et al., 2020). Unlike traditional approaches that often adopt a one-size-fits-all model, a process-based approach acknowledges the unique biopsychosocial processes underlying each individual’s experience of distress. By focusing on understanding and modifying these processes, process-based therapy offers a more tailored and effective approach to therapy. Likewise, process-based therapy, radically departs from the traditional latent disease model of psychiatry. It relies on “idionomic” network analysis, considering individuals’ life trajectories and biopsychosocial variables. Process-based therapy relies on idiographic analysis.

The path toward evidence process-based therapy must be backed by methodologies that fit it and are sensible to test its theoretical tenets. Past research on processes of change have predominantly relied on conventional methodologies, such as regression models (Hofmann et al., 2020). While these methods offer insights at the population level, they fail to capture the dynamic and bidirectional nature of individual processes of change at the person-level. Hence, these conventional approaches tend to oversimplify therapeutic phenomena and neglect the intricate interactions of patients’ psychological constructs while undergoing the therapeutic process. Also, they often overlook the diverse pathways of change among individuals, leading to incomplete understanding and inaccurate conclusions and require the acceptance of implausible assumptions regarding processes of change and the characteristics of psychotherapy as a treatment such as ergodicity, linear relationship between the variables and the outcome without changes over time, and the independence of variables from the patients’ context, through a nomothetic lens (Hayes et al., 2019; Hofmann et al., 2020).

A process-based approach assumes a more nuanced stance, incorporating mechanisms known from psychopathology and treatment literature, but applied to the individual client, thereby acknowledging the intricate interplay between various factors shaping an individual’s experience of distress and therapeutic response. This paradigm shift underscores the need for methodologies capable of capturing the richness and complexity of therapeutic change at the individual level, moving beyond the constraints of traditional statistical analyses. It is our belief that adopting a dynamic network approach holds promise for examining processes of change at the level of analysis at which the processes of change exist and can be assessed, moving from nomothetic summaries of the role of multiple variables in subgroups and populations. Thus, the call for more sophisticated methodologies becomes imperative in understanding the interconnectedness of symptoms and behaviors within individuals undergoing therapy. By embracing these advanced methodologies, researchers can gain deeper insights into the mechanisms driving therapeutic change and tailor interventions more effectively to meet the diverse needs of individuals seeking psychotherapy.

There is a growing number of protocols specifically developed to target specific constructs and processes of specific syndromes. This unbated trend should reflect an unbated progress in the fields’ knowledge. On the contrary, our knowledge regarding change processes nowadays is very limited (Kazdin et al., 2007). Randomized clinical trials have been accepted as the golden standard to assess interventions efficacy, however, given its nomothetic nature, it falls short on power to detect intra-individual processes that underlie clients’ difficulties and their change when submitted to psychotherapy. We believe that a process-based approach, with intensive and frequent assessment linked to a modern time series and network analysis can augment randomized clinical trials, fostering the research program’s sensitivity to the individual while nomothetic questions are examined, without violating logical and statistical assumptions.

The integration of process-based methodologies with randomized clinical trials offers promising avenues for advancing intervention science. By departing from solely nomothetic analyses and embracing ideographic study at the person-level, researchers can gain deeper insights into individual patterns of change and their relationship to therapeutic outcomes. This approach not only enhances our understanding of therapeutic mechanisms but also ensures that interventions are tailored to meet the unique needs of each client. In other words, a process-based approach aims to bring back the person and their idiosyncrasies, as the main object of scientific examination.

The integration of an individualized process-based approach into mental health research and practice may hold numerous positive implications for both clinicians and researchers. In the clinical setting, embracing a process-based approach capacitate therapists to customize interventions according to the unique needs of each client, improving treatment outcomes. Meanwhile, in research, studying individual processes of change offers insights that can inform the development of evidence-based interventions and theoretical models.

The transition towards a process-based approach heralds a paradigm shift in intervention science. A process-based approach has its emphasis on understanding individual change processes, allowing a deeper analysis of therapeutic interventions and psychopathology. By studying processes of change at the level of the individual, researchers can gather nomothetic summaries of individual patterns, that can be aggregated and generalized to answer to nomothetic questions at the population-level, aiming to pave way for interventions that are tailored to individual needs. Furthermore, embracing a process-based approach facilitates the transition from traditional syndrome-focused protocols to individualized idiographic approaches, by acknowledging the heterogeneity among individuals and the importance of contextual factors in therapeutic outcome. By analyzing change processes at the person-level level and then generalizing to the population-level, researchers can develop more comprehensive and personalized models of therapeutic change, that can result in more impactful and effective mental health care interventions.

## Action editor

Lars R. Bergman served as action editor for this article.

## Open access

This article is distributed under the terms of the Creative Commons Attribution 4.0 International License (http://creativecommons.org/licenses/by/4.0/), which permits unrestricted use, distribution, and reproduction in any medium, provided you give appropriate credit to the original author(s) and the source, provide a link to the Creative Commons license, and indicate if changes were made.
